# INEQUALITIES IN THE FRAILTY-FREE LIFE EXPECTANCY OF ADULTS IN ENGLAND AGED OVER 50

**DOI:** 10.1093/geroni/igad104.1793

**Published:** 2023-12-21

**Authors:** David Sinclair, Asri Maharani, Fiona Matthews

**Affiliations:** Newcastle University, Newcastle upon Tyne, England, United Kingdom; Manchester Metropolitan University, Manchester, England, United Kingdom; Newcastle University, Newcastle upon Tyne, England, United Kingdom

## Abstract

Understanding how the onset of frailty differs between socio-economic groups will help health and care providers plan for increasing demand from an ageing population and highlight population groups to target interventions to reduce frailty. We aimed to calculate the frailty-free, frail and total life expectancies of adults aged over 50 using data from the English Longitudinal Study of Ageing (ELSA), and analyse associations with socio-economic characteristics. Survey data from ELSA waves 1-9 (2002-2019) was used to follow the frailty trajectories of individuals (n=11,423 at wave 1). Individuals were categorised into non-frail and frail states using a frailty index. A multistate model assessed the risk of individuals transitioning between states or dying. Transitions were associated with the participants’ socio-economic characteristics and converted to life expectancies. Increased wealth, reduced deprivation, more education, and marriage are all associated with increased frailty-free and total life expectancies, and reduced frail life expectancies. Wealth is the most important socio-economic indicator of frailty-free life expectancy. At age 50, large inequalities in frailty-free life expectancies exist between the wealthiest, least deprived population (females: 36.1 [35.1-37.0], males: 34.6 [33.7-35.4] years) and the least wealthy, most deprived population (females: 22.1 [21.3-22.7], males: 21.3 [20.4-22.0] years). This is the first study to investigate the associations between frailty-free life expectancies and socio-economic characteristics in Europe. Large inequalities in frailty-free and frail life expectancies exist between those with different socio-economic characteristics. This highlights the people most at risk of early frailty at younger ages.

